# Patellar resurfacing in posterior cruciate ligament retaining total knee arthroplasty (PATRES): design of a randomized controlled clinical trial

**DOI:** 10.1186/1471-2474-15-358

**Published:** 2014-10-29

**Authors:** Maria JFJ Bischoff, Tom M van Raaij, Inge HF Reininga, Jos JAM van Raay

**Affiliations:** Department of Orthopaedic Surgery, Martini Hospital Groningen, Van Swietenplein 1, 9728 NT Groningen, The Netherlands

## Abstract

**Background:**

Anterior knee pain may occur after total knee arthroplasty (TKA). Patellar resurfacing, which is considered to lower the incidence of anterior knee pain after TKA, remains controversial. In the present study clinical and radiological outcomes after TKA performed on patients with clinical and radiological signs of femorotibial and patellofemoral osteoarthritis (OA) with and without patellar resurfacing will be compared.

**Methods/design:**

Fifty patients will be included in a randomized controlled trial. Patients scheduled for TKA with clinical and radiological signs of femorotibial and patellofemoral OA will be included. Arthritis of the patellofemoral joint was determined based on the preoperative Baldini and Merchant X-ray views, which is assessed by the orthopaedic surgeon who treats the patient. Exclusion criteria are rheumatoid arthritis, history of patellar fracture, tuberosity transposition, high tibial osteotomy (HTO), hip arthroplasty and posterior cruciate ligament insufficiency. Patients will be randomized to undergo TKA either with or without patellar resurfacing. Outcomes will be assessed preoperatively, at 6 weeks and at 6, 12, 18 and 24 months postoperatively. Primary outcome measure is the patellofemoral scoring system according to Baldini. Secondary outcome measures are the Knee Society clinical rating system (KSS) and the Knee Osteoarthritis Outcome Scale (KOOS) scores. Conventional weight-bearing radiographs, and views according to Baldini will be used to asses component loosening, wear, and patellofemoral problems including fracture or loosening of resurfaced patellae, subluxation and wear of non-resurfaced patellae.

**Discussion:**

There is no consensus regarding patellar resurfacing during primary TKA. Current prospective studies fail to determine any differences in clinical outcome among patients after TKA with or without patellar resurfacing. This randomized controlled trial has been designed to determine the effectiveness of patellar resurfacing during TKA in patients undergoing TKA who have clinical and radiological signs of tibiofemoral and patellofemoral OA, using a specific patellofemoral outcome measurement.

**Trial registration:**

Netherlands Trial RegistryNTR3108

**Electronic supplementary material:**

The online version of this article (doi:10.1186/1471-2474-15-358) contains supplementary material, which is available to authorized users.

## Background

Total knee arthroplasty (TKA) is a well-established surgical procedure, effective for relieving pain and improving function in patients suffering from osteoarthritis of the knee (OA)[1]. Studies have shown that more than 90% of modern primary total knee arthroplasties survive for at least ten years[2, 3]. Nonetheless, a substantial number of patients remains who have patellofemoral pain following TKA[4, 5]. Resurfacing of the patella is considered to be effective in lowering the incidence of anterior knee pain[6, 7]. Indications for patellar resurfacing during primary TKA, as described in current literature[8–11], include older age, anterior knee pain or other patellofemoral symptoms, rheumatoid arthritis (RA), obesity, history of patellar subluxation or dislocation, large and/or thick patella, a multi-operated knee joint and major loss of patellofemoral articular cartilage noted intraoperatively.

Patellar resurfacing is not without drawbacks. Complications of patellar resurfacing include patellar fracture, tendon rupture, osteonecrosis and soft-tissue impingement[12–15]. Unsatisfactory results because of patellar tilt, maltracking, instability, polyethylene wear and patellar clunk syndrome have been reported[16, 17]. Unresurfaced patellae are subjected to high compressive forces, and may develop cartilage erosion after knee joint replacement[18]. No studies found conclusive evidence that patellae affected by such changes become symptomatic after TKA[19, 20].

A meta-analysis including 1223 knees showed a 14% reduction in the absolute risk of postoperative anterior knee pain following patellar resurfacing during primary TKA (95% CI: 6–21%)[21]. By resurfacing the patella during primary TKA the risk of secondary patellar replacement was lowered by 5%[6]. Recent reports fail to demonstrate benefits of patellar resurfacing regarding functional outcome. Some reports have demonstrated that secondary patellar resurfacing after TKA leads to inferior results compared to initial patellar resurfacing during primary TKA[22–24]. Other prospective studies fail to determine any difference in clinical and functional outcome among patients after TKA with or without patellar resurfacing[8, 11, 25, 26]. A recent randomised controlled trial (RCT) including 1715 knees showed no significant difference in functional outcome between patellar resurfacing or non-resurfacing during TKA (95% CI: -0.58–1.76) using the Oxford Knee Score[27].

The inability to detect clinical differences may be caused by our lack of identifying those individuals who are thought to benefit from patellar resurfacing whilst avoiding potential complications[28]. Furthermore, current scoring systems are unable to detect subtle differences in specific patellofemoral pain and function.

The aim of the present study is to create a selective cohort of patients with clinical and radiological signs of femorotibial and patellofemoral knee OA who are indicated for TKA. All patients will be scored through the recent developed Baldini tool, a validated scoring system specifically designed to evaluate the patellofemoral joint[29, 30]. The hypothesis is that in our cohort of patients patellar resurfacing will show more than 10% improvement in the Baldini score compared to patients without patellar resurfacing 24 months after TKA. This paper reports on the study design of the PATRES (PATellar RESurfacing) trial.

## Methods and design

### Study design

The study design is a randomized controlled trial: patients will be randomly allocated to have TKA with or without patellar resurfacing. The study will be conducted at the Department of Orthopaedic Surgery of Martini Hospital Groningen, the Netherlands, a secondary referral teaching hospital. One investigator will enroll the patients. Two orthopaedic surgeons will perform the procedure, and are thus not blinded. One independent investigator, not involved with enrollment or surgical procedure, will evaluate the outcome measurements. This investigator and all patients will remain blinded through the assigned regimen. The study has been approved by a Medical Ethics Committee, and is also registered in the Netherlands Trial Registry [Reference NTRTC3108].

### Study sample

Patients suitable for enrolment in the study are candidates for TKA with clinical and radiological signs of femorotibial and patellofemoral OA. Exclusion criteria are rheumatoid arthritis, history of patellar fracture, patellar ligament transposition, high tibial osteotomy (HTO), hip arthroplasty, posterior cruciate ligament (PCL) lesion and inability to read or write the Dutch language.

### Intervention

All patients will be operated using the medial parapatellar approach and will receive a posterior cruciate ligament retaining all cemented total knee prosthesis (AGC^®^ Total Knee System, Biomet, USA). Patella denervation and osteophyte resection will be performed in all patients with or without resurfacing[9, 10, 31]. In the patellar resurfacing group the patella will be resected to the appropriate size. The posterior articulating surface will be removed with the patella everted laterally. The resection will begin just below the subchondral bone at a level, which corresponds to the thickness of the patella component to be implanted. The aim of this procedure is to ensure maximum coverage of the cut surface without implant overhang. The one-peg all-polyethylene patellar prosthesis will be cemented in one stage with the tibial and femoral components. All patients will have the same postoperative rehabilitation regime.

### Main study parameter/endpoint

Primary outcome measure is the patellofemoral scoring system by Baldini et al. This tool represents patellofemoral kinematics more accurately, and may explain related patellofemoral complaints better[30]. The scoring system includes objective and subjective aspects concerning the patellofemoral joint, ranging from 0 (worst) to 100 (best)[30].

### Secondary study parameters/endpoints

Knee function will be assessed by means of two scoring systems: the Knee Society clinical rating system (KSS) and the Knee injury and Osteoarthritis Outcome Score (KOOS). The KSS is subdivided into a knee score that rates only aspects of the knee joint itself, such as range of motion and stability, and a functional score that rates the patient’s ability to walk and climb stairs[32].The KOOS is developed as an instrument to assess patients’ opinion about their knee and associated problems[33, 34]. The KOOS consists of 5 subscales; pain, other symptoms, function in daily living, function in sport and recreation, and knee-related quality of life, with scores ranging from 0 (worst) to 100 (best).

**Figure 1:**
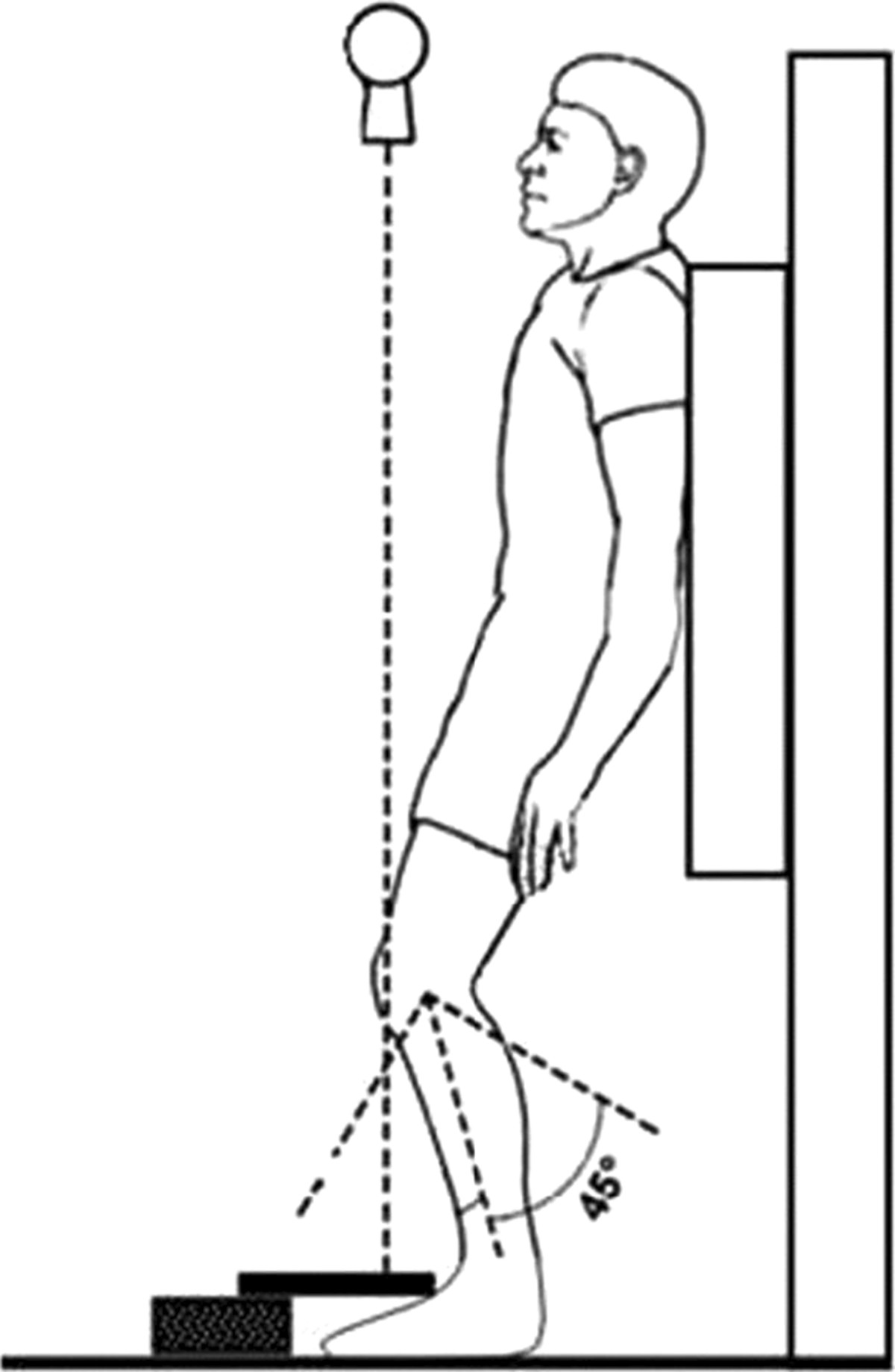
**View according to Baldini**[29]**.**

Component loosening, wear, and patellofemoral problems including fracture or loosening of resurfaced patellae, subluxation and wear of non-resurfaced patellae will be assessed on conventional weight-bearing radiographs and views according to Baldini[35]. The view according to Baldini is a dynamic weight-bearing axial radiographic view of the patellofemoral joint with the patient standing in a semi-squatting position with knees in 45 degrees of flexion (Figure 1)[29].

### Randomization

**Figure 2:**
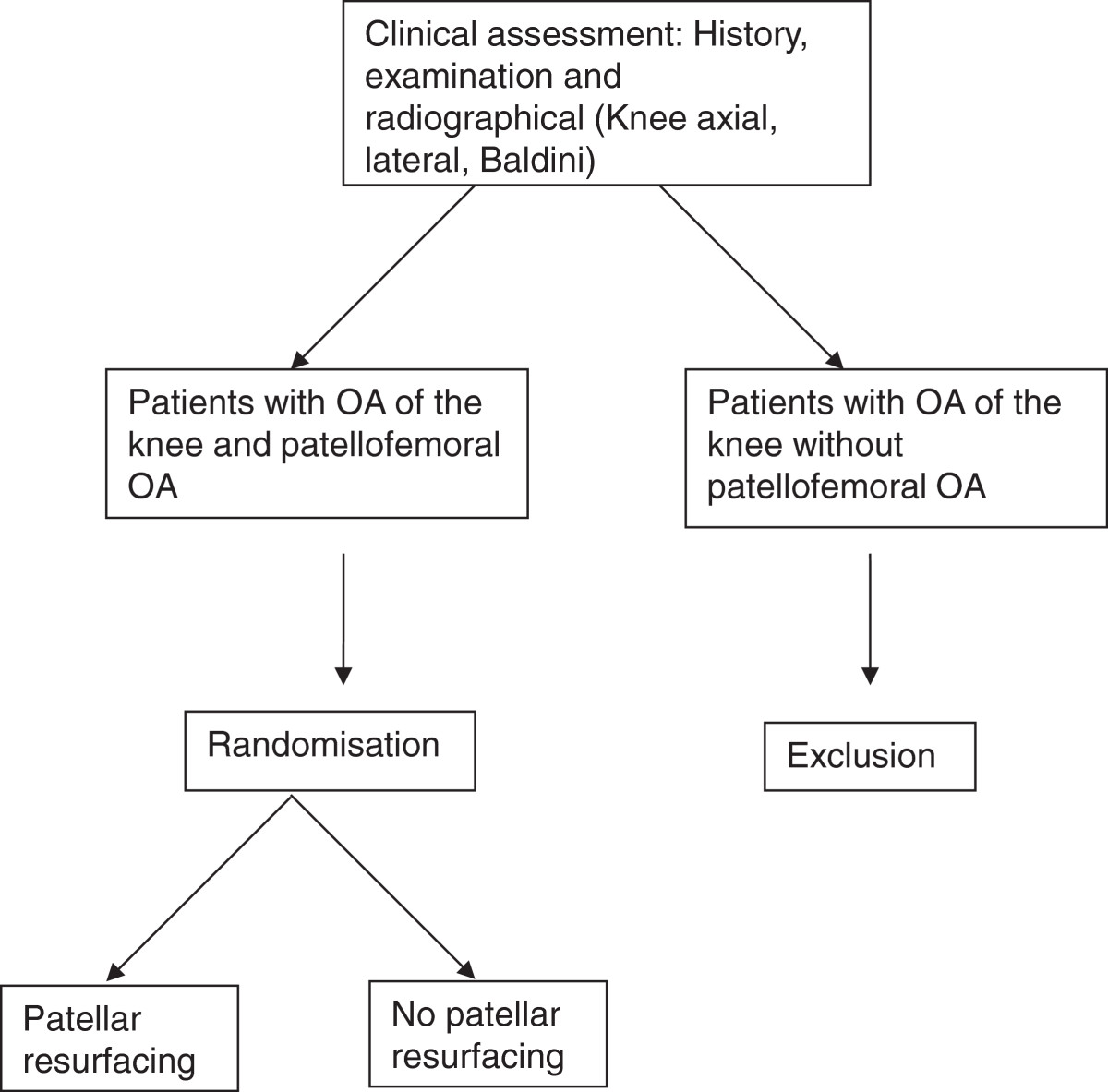
**Flowchart of patient inclusion.**

Patients who meet the inclusion criteria will be informed about the study by their orthopaedic surgeon at the Department of Orthopaedics of Martini Hospital Groningen. They will be informed about the treatment and the risk of complications according to the Dutch Medical Treatment Contracts Act. After consenting to participate, patients will be randomly assigned to one of the two regimens in a 1:1 ratio (Figure 2). The schedule for randomisation will be randomly generated using a computer before initiation of the study. To conceal the outcomes of the randomization, the allocation numbers will be put into concealed, opaque envelopes. An independent researcher will prepare the envelopes. Following informed consent, a randomization envelope will be opened. The randomisation allocation will not be notified to either the patient or the independent researcher, as both patient and investigator will be blinded to the outcome of the randomization.

### Data collection methods

Preoperatively as well as at six weeks, and six, 12,18 and 24 months postoperatively patient assessments will be performed. Age, gender, body mass index (BMI), severity of OA, will be recorded preoperatively. Perioperative complications will be registered. Primary and secondary outcome measurements will be obtained at all time points by an independent investigator (Table 1).

**Table 1:** **Data (time) management**

Follow-up	X-rays	Documentation
Day 0	Knee AP and Lateral	Basic characteristics (incl. BMI)
	Baldini	KSS/Baldini/KOOS
		Physical examination
		Informed consent
	Randomisation	
**Operation**		Standardised report
Day 1- Day 5	Knee AP and lateral	Wound check
Week 6	Knee AP and lateral	Wound check, KSS/Baldini/KOOS
	Baldini	
Month 6		KSS/Baldini/KOOS
		Physical examination
Month 12	Knee AP and lateral	KSS/Baldini/KOOS
	Baldini	Physical examination
Month 18	Knee AP and lateral	KSS/Baldini/KOOS
	Baldini	Physical examination
Month 24	Knee AP and lateral	KSS/Baldini/KOOS
	Baldini	Physical examination

### Sample size calculation

Using the patellofemoral scoring system by Baldini et al.[30], the sample size is based on a mean difference of 10 points between both groups at 2-years follow-up. To detect this difference of 10 points with a standard deviation of 8 points, alpha of 5% and a power of 90%, a sample size of 18 patients in each study group is required. To compensate for potential drop-out and loss to follow-up, a total of 50 patients will be included in the study.

### Statistical analysis

All statistical analyses will be computed using SPSS (IBM SPSS, Inc., Version 20.0, 2011). For the clinical parameters, t-tests will be used for continuous variables or the Mann–Whitney U-test when the variables are not normally distributed. A Chi-square test will be used for dichotomous variables. To assess differences in the results of the Baldini score and the KOOS over time between the two study groups, generalised estimating equations (GEE) analyses will be conducted. Longitudinal data are characterised by repeated observations of the same subjects. GEE analysis was developed to correct for repeated outcomes within the same subject[36]. With a GEE analysis, adjustments for the effect of differences in patient characteristics on the outcome variables can also be made by including these variables as covariates. Both an intention-to-treat analysis as a per-protocol analysis will be performed.

## Discussion

The need to resurface the patella as part of a total knee arthroplasty (TKA) remains controversial[12, 37, 38]. Several prospective observational studies have shown that approximately 10% of patients are affected by significant patellofemoral complaints after TKA, despite patellar resurfacing[39–41].

Selective resurfacing may improve clinical success by identifying individuals who will benefit from resurfacing the patella[28]. In the present study all the included patients will have symptomatic patellofemoral OA as well as femorotibial OA. In our opinion this selection of patients provides a cohort that enables us to draw valid conclusions on patellar resurfacing with the use of the AGC total knee system. At our department the AGC^®^ Total Knee System is used. This knee prosthesis has excellent longevity with minimal wear despite a flat-on-flat geometry and posterior cruciate ligament retainment[42, 43]. Emerson et al.[43] showed a low rate of patellar failure in the AGC total knee system. Favourable component alignment together with unconstrained patellofemoral articulation most likely minimises patellofemoral stress. However, no specific patellofemoral scoring system was used in this study.

Baldini et al.[30] published a scoring system specifically designed to evaluate the patellofemoral joint before and after TKA. This scoring system is developed to determine function and pain, ranging from 0 to 100 points, and includes ratings for subjective and objective clinical aspects of the patellofemoral joint before and after TKA[30]. This dedicated patellofemoral scoring system will be used in our clinical trial to improve the accuracy of the clinical outcome assessment. Feller et al. performed a prospective study with a self-made specific score for the patellofemoral joint and arthritis. No significant difference between the two treatment groups (resurfacing versus non-resurfacing) for their patellar score was found. Although the study of Feller et al. has a similar design as the current study, they did not use a homogenous patient group: by contrast, in the present study all the included patients will have clinical and radiological femorotibial and patellofemoral OA[31].

The use of patient selection and a specific validated measurement tool allow us to expect to find a significant difference between the two groups that other prospective trials could not.

Most of these studies use established clinical knee scoring systems, such as the KSS[32] and the KOOS[33] as outcome assessments. The KSS is derived from clinical and radiological data and concentrates mainly on the surgeon’s view of the outcome, i.e. knee pain, joint deformity and knee motion[44–46]. Patients are more concerned with symptoms and functional limitations. It is well recognised that clinical outcome assessments and patient-related measurements evaluate different aspects of knee injury and knee OA. Weak correlations and frequent discordance are found when comparing clinical outcome assessments like radiographic findings and knee motion to patient-relevant outcomes such as pain, function and activity level[47–50].

A patient-reported outcome measure, specific for knee problems, will thus be used in this study. The KOOS used in our clinical trial is developed to assess patients' opinion about their knee and related problems[33]. The KOOS is a comprehensive instrument that includes five subscales assessing aspects of knee injury and knee OA considered important by patients. Most other instruments used for knee functional status combined items measuring different aspects into one score.

An additional aspect in studies assessing outcome of TKA is a discrepancy between patellofemoral symptoms and results obtained by conventional scoring and radiographic analysis[47–49]. Regular scoring systems focus mainly on tibiofemoral aspects and specific patellofemoral symptoms can be missed or underscored. Commonly used clinical outcome measures, such as the KSS, do not take into account subjective symptoms or objective data specifically arising from the patellofemoral joint. The Baldini score represents an essential complementary source of information to the existing KSS and KOOS scores since both scores do not specifically focus on patellofemoral problems.

Our study design is limited that results may not be applicable to other knee systems; especially posterior stabilized (PS) TKA. Alternative knee replacement designs may affect patellar kinematics differently. An in vitro study, however, showed similar quadriceps forces using CR or PS TKA systems[51]. To our knowledge no clinical trials have been published to confirm these findings.

Previous randomised controlled trials on the effectiveness of patellar resurfacing during primary TKA have included patients with knee OA regardless of patellofemoral complaints. This trial studies a selected cohort of patients receiving total knee replacement. All included patients have clinical and radiological patellofemoral OA besides symptomatic femorotibial OA. This is a difference compared to previous randomised controlled trials that have included patients with knee OA regardless of patellofemoral complaints.

Radiographic assessments used in clinical trials to evaluate the patellofemoral joint after TKA have remained basically unchanged for a number of decades, and do not accurately assess dynamic patellofemoral tracking following arthroplasty[52, 53]. Traditional radiographic assessment in a static unloaded position may not reproduce in vivo patellofemoral kinematics[54]. On the commonly used tangential patellar axial view radiographs, the patellar position relative to the trochlea is independent of fundamental dynamic and neuromuscular factors[55, 56]. The role of the quadriceps muscle in affecting the position of the patella relative to the femur must be considered. Additionally, patellar kinematics are influenced by the axial and rotational alignment of the lower limb[57]. Patients with patellofemoral problems following TKA typically experience anterior knee pain when the extensor apparatus is under load. Baldini et al.[30] developed a dynamic weight-bearing radiographic view that reproduces a semi-squatting positional setting which sufficiently loads the patellofemoral joint and involves the extensor apparatus. This view according to Baldini will be used in our clinical trial because it allows for better assessment of patellofemoral OA and patellar subluxation[29].

## Conclusion

This paper describes a novel the design of a randomized controlled clinical trial on patellar resurfacing during TKA. A selected group of patients and the use of a specific patellofemoral scoring system will allow us to identify patients who will benefit from patellar resurfacing more accurately[29, 30]. This study can contribute to the decision making of resurfacing or not in primary TKA.

## Authors’ original submitted files for images

Below are the links to the authors’ original submitted files for images. Authors’ original file for figure 1 Authors’ original file for figure 2

## References

[1] Merle-Vincenta F (2010). Factors predicting patient satisfaction 2 years after total knee arthroplasty for osteoarthritis. Joint Bone Spine.

[2] Rodriguez JA, Bhende H, Ranawat CS (2001). Total condylar knee replacement: a 20-year followup study. Clin Orthop Relat Res.

[3] Mont MA, Pivec R, Issa K, Kapadia BH, Maheshwari A, Harwin SF (2013). Long-term implant survivorship of cementless total knee arthroplasty: a systematic review of the literature and meta-analysis. J Knee Surg.

[4] Castro FP, Chimento G, Munn BG, Levy RS, Timon S, Barrack RL (1997). An analysis of Food and Drug Administration medical device reports relating to total joint components. J Arthroplasty.

[5] Parker DA, Dunbar MJ, Rorabeck CH (2003). Extensor mechanism failure associated with total knee arthroplasty: prevention and management. J Am Acad Orthop Surg.

[6] Waters TS, Bentley G (2003). Patellar resurfacing in total knee arthroplasty - A prospective, randomized study. J Bone Joint Surg Am.

[7] Tabutin J, Banon F, Catonne Y, Grobost J, Tessier JL, Tillie B (2005). Should we resurface the patella in total knee replacement? Experience with the Nex Gen prothesis. Knee Surg Sports Traumatol Arthrosc.

[8] Burnett RS, Haydon CM, Rorabeck CH, Bourne RB (2004). Patella resurfacing versus nonresurfacing in total knee arthroplasty - Results of a randomized controlled clinical trial at a minimum of 10 years' followup. Clin Orthop.

[9] Nizard RS, Biau D, Porcher R, Ravaud P, Bizot P, Hannouche D, Sedel L (2005). A meta-analysis of patellar replacement in total knee arthroplasty. Clin Orthop.

[10] Patel K, Raut V (2011). Patella in total knee arthroplasty: to resurface or not to-a cohort study of staged bilateral total knee arthroplasty. Int Orthop.

[11] Smith AJ, Wood DJ, Li MG (2008). Total knee replacement with and without patellar resurfacing - A prospective, randomised trial using the profix total knee system. J Bone Joint Surg Br.

[12] Ranawat CS (1986). The patellofemoral joint in total condylar knee arthroplasty - pros and cons based on 5-year to 10-year follow-up observations. Clin Orthop.

[13] Webster DA, Murray DG (1985). Complications of variable axis total knee arthroplasty. Clin Orthop.

[14] Grace JN, Rand JA (1988). Patellar instability after total knee arthroplasty. Clin Orthop.

[15] Ortiguera CJ, Berry DJ (2002). Patellar fracture after total knee arthroplasty. J Bone Joint Surg Am.

[16] Hozack WJ, Rothman RH, Booth RE, Balderston RA (1989). The patellar clunk syndrome - a complication of posterior stabilized total knee arthroplasty. Clin Orthop.

[17] Fehring TK, Odum S, Griffin WL, Mason JB, Nadaud M (2001). Early failures in total knee arthroplasty. Clin Orthop.

[18] Forster H, Fisher J (1999). The influence of continuous sliding and subsequent surface wear on the friction of articular cartilage. Proc Inst Mech Eng H.

[19] Keblish PA, Varma AK, Greenwald AS (1994). Patellar resurfacing or retention in total knee arthroplasty. A prospective study of patients with bilateral replacements. J Bone Joint Surg (Br).

[20] Soudry M, Mestriner LA, Binazzi R, Insall JN (1986). Total knee arthroplasty without patellar resurfacing. Clin Orthop Relat Res.

[21] Pakos EE, Ntzani EE, Trikalinos TA (2005). Patellar resurfacing in total knee arthroplasty - A meta-analysis. J Bone Joint Surg Am.

[22] Clements WJ, Miller L, Whitehouse SL, Graves SE, Ryan P, Crawford RW (2010). Early outcomes of patella resurfacing in total knee arthroplasty A report from the Australian Orthopaedic Association National Joint Replacement Registry. Acta Orthop.

[23] Khatod M, Codsi M, Bierbaum B (2004). Results of resurfacing a native patella in patients with a painful total knee arthroplasty. J Knee Surg.

[24] Muoneke HE, Khan AM, Giannikas KA, Hagglund E, Dunningham TH (2003). Secondary resurfacing of the patella for persistent anterior knee pain after primary knee arthroplasty. J Bone Joint Surg Br.

[25] Wood DJ, Smith AJ, Collopy D, White B, Brankov B, Bulsara MK (2002). Patellar resurfacing in total knee arthroplasty - A prospective, randomized trial. J Bone Joint Surg Am.

[26] Barrack RL, Bertot AJ, Wolfe MW, Waldman DA, Milicic M, Myers L (2001). Patellar resurfacing in total knee arthroplasty - A prospective, randomized, double-blind study with five to seven years of follow-up. J Bone Joint Surg Am.

[27] Breeman S, Campbell M, Dakin H, Fiddian N, Fitzpatrick R, Grant A, Gray A, Johnston L, Maclennan G, Morris R, Murray D, KAT Trial Group (2011). Patellar resurfacing in total knee replacement: five-year clinical and economic results of a large randomized controlled trial. J Bone Joint Surg Am.

[28] Schindler OS (2012). The controversy of patellar resurfacing in total knee arthroplasty: Ibisne in medio tutissimus?. Knee Surg Sports Traumatol Arthrosc.

[29] Baldini A, Anderson JA, Cerulli-Mariani P, Kalyvas J, Pavlov H, Sculco TP (2007). Patellofemoral evaluation after total knee arthroplasty - Validation of a new weight-bearing axial radiographic view. J Bone Joint Surg Am.

[30] Baldini A, Anderson JA, Zampetti P, Pavlov H, Sculco TP (2006). A new patellofemoral scoring system for total knee arthroplasty. Clin Orthop.

[31] Feller JA, Bartlett RJ, Lang DM (1996). Patellar resurfacing versus retention in total knee arthroplasty. J Bone Joint Surg Br.

[32] Insall JN, Dorr LD, Scott RD, Scott WN (1989). Rationale of the knee-society clinical rating system. Clin Orthop.

[33] Roos EM, Roos HP, Lohmander LS, Ekdahl C, Beynnon BD (1998). Knee Injury and Osteoarthritis Outcome Score (KOOS)–development of a self-administered outcome measure. J Orthop Sports Phys Ther.

[34] de Groot IB, Favejee MM, Reijman M, Verhaar JA, Terwee CB (2008). The Dutch version of the knee injury and osteoarthritis outcome score: a validation study. Health Qual Life Outcomes.

[35] Ewald FC (1989). The knee-society total knee arthroplasty roentgenographic evaluation and scoring system. Clin Orthop.

[36] Twisk JW (2003). Applied Longitudinal Data Analysis for Epidemiology.

[37] Aglietti P, Buzzi R, De Felice R, Giron F (1999). The Insall-Burstein total knee replacement in osteoarthritis - A 10-year minimum follow-up. J Arthroplasty.

[38] Berger RA, Rosenberg AG, Barden RM, Sheinkop MB, Jacobs JJ, Galante JO (2001). Long-term followup of the Miller-Galante total knee replacement. Clin Orthop.

[39] Brander VA, Stulberg SD, Adams AD, Harden RN, Bruehl S, Stanos SP, Houle T (2003). Predicting total knee replacement pain: a prospective, observational study. Clin Orthop Relat Res.

[40] Elson DW, Brenkel IJ (2006). Predicting pain after total knee arthroplasty. J Arthroplasty.

[41] Heck DA, Robinson RL, Partridge CM, Lubitz RM, Freund DA (1998). Patient outcomes after knee replacement. Clin Orthop Relat Res.

[42] Ritter MA, Berend ME, Meding JB, Keating EM, Faris PM, Crites BM (2001). Long-term followup of anatomic graduated components posterior cruciate-retaining total knee replacement. Clin Orthop Relat Res.

[43] Emerson RH, Higgins LL, Head WC (2000). The AGC total knee prosthesis at average 11 years. J Arthroplasty.

[44] Franke KF, Nusem I, Gamboa G, Morgan DA (2013). Outcome of revision total knee arthroplasty with bone allograft in 30 cases. Acta Orthop Belg.

[45] Lutzner J, Hartmann A, Lutzner C, Kirschner S (2013). Is range of motion after cruciate-retaining total knee arthroplasty influenced by prosthesis design? A prospective randomized trial. J Arthroplasty.

[46] Pilling RW, Moulder E, Allgar V, Messner J, Sun Z, Mohsen A (2012). Patellar resurfacing in primary total knee replacement: a meta-analysis. J Bone Joint Surg Am.

[47] Hadler NM (1992). Knee pain is the malady–not osteoarthritis. Ann Intern Med.

[48] Lethbridge-Cejku M, Scott WW, Reichle R, Ettinger WH, Zonderman A, Costa P, Plato CC, Tobin JD, Hochberg MC (1995). Association of radiographic features of osteoarthritis of the knee with knee pain: data from the Baltimore Longitudinal Study of Aging. Arthritis Care Res.

[49] Cicuttini FM, Baker J, Hart DJ, Spector TD (1996). Association of pain with radiological changes in different compartments and views of the knee joint. Osteoarthritis Cartilage.

[50] Snyder-Mackler L, Fitzgerald GK, Bartolozzi AR, Ciccotti MG (1997). The relationship between passive joint laxity and functional outcome after anterior cruciate ligament injury. Am J Sports Med.

[51] Heyse TJ, Becher C, Kron N, Ostermeier S, Hurschler C, Schofer MD, Fuchs-Winkelmann S, Tibesku CO (2010). Quadriceps force in relation of intrinsic anteroposterior stability of TKA design. Arch Orthop Trauma Surg.

[52] Bindelglass DF, Cohen JL, Dorr LD (1993). Patellar tilt and subluxation in total knee arthroplasty - relationship to pain, fixation, and design. Clin Orthop.

[53] Laughlin RT, Werries BA, Verhulst SJ, Hayes JM (1996). Patellar tilt in total knee arthroplasty. Am J Orthop (Belle Mead NJ).

[54] Katchburian MV, Bull AM, Shih YF, Heatley FW, Amis AA (2003). Measurement of patellar tracking: assessment and analysis of the literature. Clin Orthop Relat Res.

[55] Merchant AC, Mercer RL, Jacobsen RH, Cool CR (1974). Roentgenographic analysis of patellofemoral congruence. J Bone Joint Surg Am.

[56] Laurin CA, Levesque HP, Dussault R, Labelle H, Peides JP (1978). The abnormal lateral patellofemoral angle: a diagnostic roentgenographic sign of recurrent patellar subluxation. J Bone Joint Surg Am.

[57] Lee TQ, Morris G, Csintalan RP (2003). The influence of tibial and femoral rotation on patellofemoral contact area and pressure. J Orthop Sports Phys Ther.

[CR58] The pre-publication history for this paper can be accessed here:http://www.biomedcentral.com/1471-2474/15/358/prepub

