# Supplementary figures and images for: Patellar resurfacing in posterior cruciate ligament retaining total knee arthroplasty (PATRES): design of a randomized controlled clinical trial
Source: BMC Musculoskelet Disord. 2014 Oct 29;15:358. doi: 10.1186/1471-2474-15-358 (PMC4232658; doi:10.1186/1471-2474-15-358)

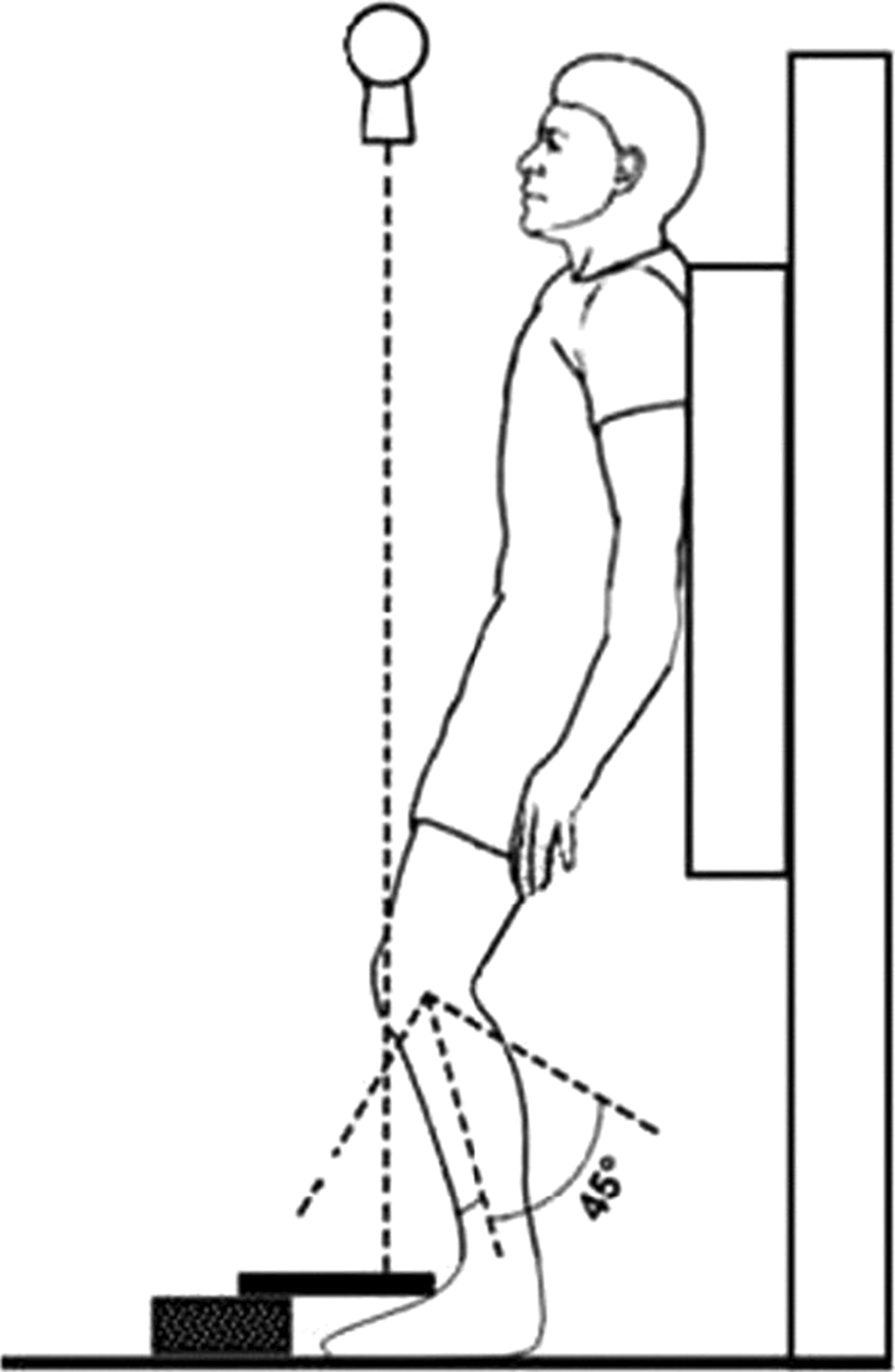

Supplement: Supplementary file 1 — Authors’ original file for figure 1 [file 12891_2013_2302_MOESM1_ESM.tif]
