# Supplementary material for: Patellar resurfacing in posterior cruciate ligament retaining total knee arthroplasty (PATRES): design of a randomized controlled clinical trial
Source: BMC Musculoskelet Disord. 2014 Oct 29;15:358. doi: 10.1186/1471-2474-15-358 (PMC4232658; doi:10.1186/1471-2474-15-358)
Supplement: Supplementary file 2 — Authors’ original file for figure 2 [file 12891_2013_2302_MOESM2_ESM.pdf]

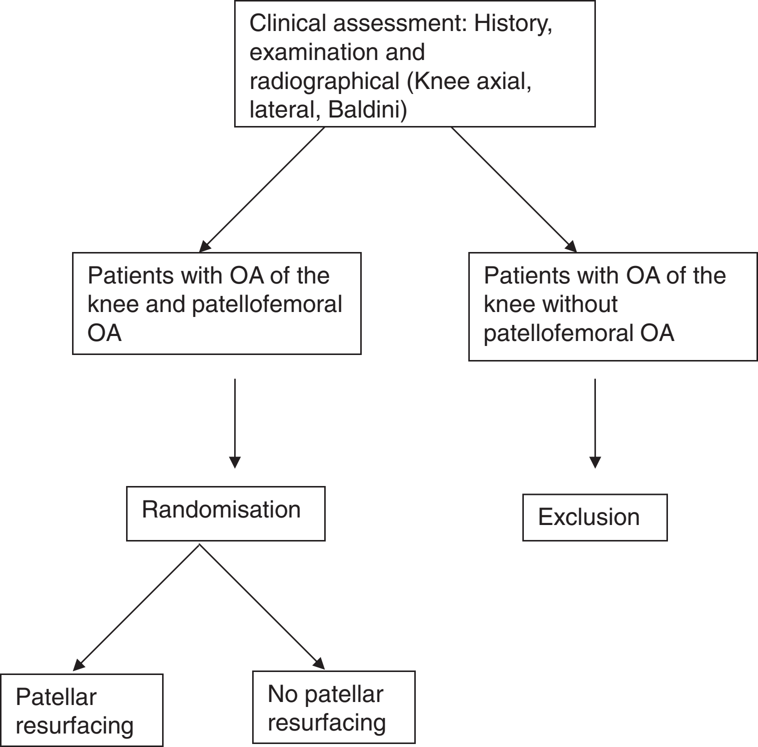
Clinical assessment: History, examination and radiographical (Knee axial, lateral, Baldini)
Patients with OA of the knee and patellofemoral OA
Patients with OA of the knee without patellofemoral OA
Randomisation
Exclusion
Patellar resurfacing
No patellar resurfacing
